# The Valuation of Some Enteric Fever Symptoms

**Published:** 1911-07

**Authors:** Rufus T. Dorsey

**Affiliations:** Prof. Clinical Medicine, Atlanta School of Medicine


### THE VALUATION OF SOME ENTERIC FEVER SYMPTOMS.

By Rufus T. Dorsey, B.S., M.D.

*Prof. Clinical Medicine, Atlanta School of Medicine.*

The Committee's assignment of symptoms was altogether too liberal to be thoroughly presented by me within the allotted time and it is understood that I make no attempt to deal comprehensively with the subject. Therefore, a few selected symptoms and complications only will be brought to your attention for consideration.

I have a just and high appreciation of laboratory methods concerning typhoid and offer not one iota of disparagement of the sometimes valuable Widal, the negatively pathognomonic Diazo, the positively pathognomonic isolation of bacillus typhosus, and the occasional invaluable leucopenia and hypertension determination, but I would like to emphasize to the bedside student that these, with few exceptions, are unnecessary for accurate work, provided he is thoroughly and immediately familiar, both theoretically and clinically, with the symptomatology of typhoid fever. No accessory methods, however definite, can usurp or render superfluous a good, common-sense analysis and interpretation of symptoms in this disease. I insist on the enlistment of laboratory aids in obscure cases and when in doubt, but more do I insist that doubt would be less frequent if we better understood our symptomatology. Some practitioners look on the laboratory as a diagnostic mill and as exempting them from further investigations of symptoms in given cases after receiving the laboratory report. Appreciate and utilize all true aids when ad visable, at the same time, recognize that the essential thing is a proper knowledge and estimation of individual symptoms, and even more so of the group signs, as much comibnation will reduce error to a minimum. It certainly would be worth while.

### Fever.

The stepladder temperature is a particularly valuable and characteristic symptom when obtained, but it is so often robbed of its symmetry by remedial measures that stress should not be laid on the existence of an atypical type in ruling out enteric. The cone-shaped chart or short gastigium means a diminished virulence of organisms and a mild infection. The inverse type, a high morning and low evening, is authentically rare and of no importance. I have no doubt many reported cases, unless due to therapy, resulted from unsuspected phthisis. The only one I ever saw chagrined me by developing an hemoptisis and subsequently showing organisms in the sputum. In children, suddenly developed high fever ushering in typhoid is not so rare and should not mislead, and a fever of 105 persisting for three days is of ill omen. Sudden and pronounced fall of temperature is nearly always of grave import and indicates hemorrhage, perforation or grave collapse. Marked morning remissions are often the first indications of a tendency to recover and are reliable. In convalescence the fever of relapse and recrudescence can be early distinguished, except in few instances, by careful study of the symptoms which time prevents my entering into. With a knowledge that biliary and intestinal stasis cause more relapses and that they, as a rule, are easily preventable, the occasions for ex ercising discrimination would seldom arise.

GURGLING & TENDERNESS.

Gurgling and tenderness in right ilias fossa are altogether lacking in many cases and is a symptom much overated, being found in nearly all conditions characterized by intestinal flux. It indicates only the presence of flued feces and gas in the cecum and colum. Early tenderness is decidedly more suggestive of appendix involvement than of typhoid fever. They are symptoms of little importance and aid to me.

DIARRHOEA.

Diarrhoea is a variable symptom. I believe it to be much less common and less severe than formerly. It seems not to be the factor that it was even five years ago. Occasionally a severe diarrhoea depletes and undermines the patient and invites complications, but as a rule, its presence or absence is not a reliable index as to the exten of the intestinal ulceration or of the ultimate outcome. Constipation is a prevalent symptom. In 147 cases treated in the last five years diarrhoea was in the decided minority. When diarrhoea is present in typhoid the stools are usually copious in quantity, grayish yellow in color and pea soup in consistence, but these qualities do not distinguish them as typhoidal stools, as some have concluded. Stools giving these peculiarities are to be witnessed in other maladies. The characteristics of a typhoid stool, if there be any of diagnostic weight, is the strong alkalinity, coupled with strong, offensive, musty odor, impossible to describe and learned only by actually smelling. Ocular examination alone will not suffice to pass judgement on this symptom, but it is sometimes very valuable in detecting the blood tinged stools which precede and forewarm of a profuse hemorrhage.

### Odor.

A peculiar and characteristic exhalation from breath and skin is met with and should receive a limited diagnostic valuation. It is not as some believe a distinctive odor of typhoid, though certainly worth while. It persists through the illness and is not eradicated by bathing or sponging.

### Vomiting.

Except in childhood and under inappropriate medication, vomiting is unusual. When it occurs early and is rebellious, a severe course may be prophesied. When it develops late, untoward developments are likely to show up in a great majority of instances. The latter statement is rarely ever to be qualified.

### Meteorism.

Meteroism is less constant in children than in adults and is a symptom of the greatest importance, not for diagnosis but in prognosis. In my cases, flat, soft abdomens meant mild cases and few complications. In decided tympany the opposite almost invariably. While meteorism results secondarily from intesinal ulceration and toxemia and converse is truer. Excessive tympany is due to bowel paresis and mechanically embarresses the heart and pulmonic excursions, stretches the intestines, and directly favors hemorrhage, perforation and toxemia. A proper treatment of this one symptom mitigates the whole.

### Rose Spots.

The rose spots varying from single to many are found in 70 per cent of all cases about the end of the first week. These little spots are entitled to more diagnostic and prognostic esteem than is commonly accorded—though children often develop no or very slight eruption. The more numerous the rash and successive crops the more pronounced the infection holds good on more than a majority of all cases. My old professor, J. C. Wilson of Jefferson, believes copious eruption bespeaks more liberality to hemorrhage. If these statemnts are only partially true this little sign contributes materially and time spent in its consideration will be amiss only to those thoroughly versed. Disregarding other symptoms, to say with conclusive confidence that a given spot is a rose spot, is not so easy. Rose spots are only infrequently found in considerable numbers and are located generally over abdomen, but it is not unusual to find them on back, chest and rarely on face. They last only a few days and recur in groupes. Rose spots are hyperemic very slightly elevated, though palpable, pale red pimples, vanishing on touch and tension and instantly reappearing when same is removed, averaging in size a pin head. They are smaller than pimples, a fainter rose color and attract less attention in that they possess more poorly defined edges. They are further differentiated from ache by presenting an abtuse point and not a well defined conical center in developing independent of hair follicles and cutaneous glands in never suppurating and very seldom in being tipped by a vesicle. Their duration is shorter and a minimum or no stain at all ot the skin is to be discerned. A skin pencil serves me well in the observation of these spots. The genuine rose spots very infrequently occur in conditions where organisms are circulating in the blood. This fact, coupled with their inconstancy, only prevents their being an absolutely pathognomonic symptom at an opportune time.

### Circulatory System.

As to the Circulatory System, it presents no pathogomonic features, but the character and frequency of the pulse in relation to fever are very significant. The rate is increased, but not proportionately, to the common ratio of 10 to 1. The early dicrotic pulse so regularly and apparently easy to determine by many, is to me worth little in a run of cases, because I confess my inability to be positively certain that dicrotism is really there. When the slower pulse later changes to the double pulse, we have a symptomatic point worth much attention. Dicrotism is simply due to the loss of tension in the arterial wall and is said to foretell hemorrhage is usually pronounced. A pulse of 20 habitually maintained, (Like "Hardy") always gives me concern and nearly always trouble results.

### Blood Pressure.

Blood pressure is ordinarily low and varies fairly constantly with the severity of the attack. A decided progressive lowering of pressure indicates an unsatisfactory progress, and a quick loss of peripheral resistance points strongly towards hemorrhage, visible or concealed. Pressure should be determined with more regularity than is commonly done as it may reveal good data. Cold water applications increase readings from 5 to 25 m. m. and this is one of the many little ways that hydrotherapy benefits.

Leucopenia or more accurately no leucocytosis, is an excellent diagnostic symptom and is seldom lacking in the absence of septic and inflammatory additions. The white count is of prime importance in doubtful perforation.

### Splenic Enlargement.

Acute splenic enlargement to a greater or less degree, is a constant and important sign. It is demonstrable by proper palpation much more readily than by untrustworthy percussion, especially in distended abdomens and in the highly nervous. If they really feel the spleen, I am jealous of those examiners who simply and quickly dig their fingers under the costal margin and say "spleen enlarged," for they have acquired a skill which I have long since lost hope of ever attaining. With me such hooking immediately excites such muscular contractions that rigid muscles is all that I can possibly feel. I have often wished to see some palpating artist examine a patient on whom splenectomy had been performed. In order for me to satisfactorily determine the moderate splenic swelling as is usually seen in typhoid, I must place my patient in a reaxed dorsal decubitus and my palpating hand smoothly oved the left hypochondrium and then gradually exert more and more uniform pressure at each expiration until reward by touching the inferior border of the spleen at end of deep inspiration. In very young children and when enteroptosis is present, we are misled unless caution in diagnosing the size is displayed. It is a finding of A-1 importance and with history is extremely valuable.

### Renal System.

I have nothing worth while to say concerning renal symptoms or complications and only remind you that the onset of a few cases now and then is concealed by alarmingly nephritic symptoms, for which the designation nephro-typhoid is entirely appropriate.

### Respiratory Symptoms.

Concerning the respiratory system, I would call your attention to bronchitis alone. The existence of a low grade bronchitis and often in the prodromal stage is the rule—chiefly insignificant and overlooked, it is evident only by occasional cough and a few scattered sibilant rales. This symptom, by reason of this constancy, is scarcely of secondary importance, though of no gravity.

### Nervous System.

There is nothing characteristic in the nervous symptoms of typhoid, though marked symptoms express the severity generally. The prodromal insomnia, headache and mental habitude, etc., occur in other diseases. However, if the headache is persistent, tends to become worse as the night advances, ceases spontaneously, being replaced by apathy and stupor, it becomes a very much worthwhile symptom. In only fully developed, grave cases where there is an almost overwhelming infection with virulent organisms, do we nowadays have the more ominous nervous symptoms. Carphologia, subsultus tendinum, delirium and coma vigil are rarely encountered of recent years. I blieve they should be classified as complications instead of symptoms and I further believe are preventable is most cases. Hydrotherapy has worked wonders in this regard, but to that good eliminant, old fashioned calomel, should go the blue ribbon. I have had a surprisingly small percentage in my cases. These symptoms, together with what we call toxemia, are more than fairly proportionate to intestinal stasis and meteorism, and calomel judicially used will largely control them and preclude the others. The truth in the ahporism of Hippocrates that "Experience is fallacious and judgment difficult," is the only thing that deters me from swearing that it is the greatest remedy on earth for typhoid fever.

Postfebrile mania is of scarcely any gravity. I have never personally heard of but one case that failed to recover and by inheritance he was predisposed. I often wonder if undernutrition is not the sole cause and if the Coleman diet would not prevent this annoying sequel.

### PERFORATION.

Perforation occurs in 3 per cent of all cases and is a complication of gravest import. While it infrequently happens in apparently mild types, it is more common in severer cases. It is prone to develop in cases showing marked meteorism or where hemorrhage has previously presented. Never have I personally had or seen a perforation unaccompanied by marked tympanites. The case may be unusually severe as regards its other manifold symptoms, but so long as the abdomen is little distended, I little concern myself about a probable perforation or hemorrhage. This attitude may be wrong but in the light of my past experience, I believe it to be true. Recent surgical statistics in typhoid perforation show extremely gratifying results prior to the onset of actual peritonitis. Let us then study diligently the resulting symptoms—make early diagnosis, and give our patients this surgical chance. To date, I am constrained to admit that my incompetence is conspicuous, as all my diagnosis of perforation were made too late, according to the operating surgeon. If we remember that about 95 per cent of perforations are within two feet of the ileocecal valve, valuable time will be saved, at the time of intervention.

